# Off-treatment bone mineral density changes in postmenopausal women receiving anastrozole for 5 years: 7-year results from the IBIS-II prevention trial

**DOI:** 10.1038/s41416-020-01228-2

**Published:** 2021-01-22

**Authors:** Ivana Sestak, Glen Blake, Raj Patel, Jack Cuzick, Anthony Howell, Robert Coleman, Richard Eastell

**Affiliations:** 1 Centre for Cancer Prevention, Wolfson Institute of Preventive Medicine, Queen Mary University London, Charterhouse Square, London, EC1M 6BQ UK; 2 School of Biomedical Engineering & Imaging Sciences, King’s College London, St Thomas’ Hospital, London, SE1 7EH UK; 3 Imperial College London, London, SW7 2AZ UK; 4 Division of Cancer Sciences, University of Manchester, Manchester, M13 9PL UK; 5 Department of Oncology and Metabolism, University of Sheffield, Western Bank, Sheffield, S10 2TN UK

**Keywords:** Breast cancer, Randomized controlled trials

## Abstract

**Background:**

Anastrozole has been associated with substantial accelerated bone mineral density (BMD) loss during active treatment.

**Methods:**

One thousand four hundred and ten women were included in a BMD substudy and stratified into three strata according to their baseline T-score at spine or femoral neck. The primary objective of this analysis was to investigate whether DXA BMD at the spine and hip changed two years after treatment cessation (between years 5 and 7) in those who did not receive risedronate.

**Results:**

Five- and seven-year BMD data were available for a total of 528 women who did not receive risedronate. In women with normal BMD at baseline, an increase in BMD at the lumbar spine after anastrozole withdrawal was observed 1.25% (95% CI 0.73 to 1.77) (*P* = 0.0004), which was larger than in those on placebo (0.14% (−0.29 to 0.56))). At the hip, BMD remained unchanged between years 5 and 7 for those previously on anastrozole but continued to a decrease in those who had been randomised to placebo (−1.35% (−1.70 to −0.98)).

**Conclusions:**

These are the first results reporting BMD changes after stopping anastrozole in a breast cancer prevention setting. Our results show that the negative effects of anastrozole on BMD in the preventive setting are partially reversible.

## Background

Aromatase inhibitors are the choice of adjuvant endocrine treatment in postmenopausal women with early oestrogen receptor positive breast cancer breast cancer. They have shown superior efficacy and a better safety profile over tamoxifen in the adjuvant setting.^1–3^ In the prevention setting, the MAP.3 trial^4^ investigated exemestane compared to placebo to reduce the risk of developing breast cancer and reported significant bone mineral density (BMD) loss at both the lumbar spine and total hip after 2 years of follow-up on exemestane compared to placebo.^5^ Overall, no increases in fractures with exemestane were observed, but the follow-up of 35 months was too short for firm conclusions concerning this endpoint.

The International Breast cancer Intervention Study (IBIS-II) trial compared anastrozole with placebo in postmenopausal women at high risk of developing breast cancer. A significant 53% reduction in breast cancer with anastrozole was found,^6^ with updated results showing a continued effect of anastrozole with longer follow-up.^7^ A substudy of the IBIS-II trial investigated the effect of risedronate on bone density and measured BMD regularly in all participants included in this study.^8^ An initial analysis of the BMD data confirmed a significant decrease in BMD at the lumbar spine and total hip in women who were randomised to anastrozole when compared to placebo.^8^ However, a significant improvement in anastrozole-induced BMD loss at both sites was observed for women with osteopenia and osteoporosis who were taking risedronate. An updated analysis of the 5-year data showed that in osteopenic women risedronate counterbalances the negative effect of anastrozole at the lumbar spine but not the total hip.^9^ These updated results suggest that risedronate is most active and beneficial in the first 2 years of treatment with regards to preventing anastrozole-induced bone loss.

It is important to understand the effects of long-term aromatase inhibitor therapy on changes in BMD in the preventive setting in more detail. Here, we analysed the changes in BMD between 5 and 7 years after randomisation with the primary aim to evaluate the recovery of BMD after stopping anastrozole when given alone (without risedronate). A secondary aim was to investigate BMD recovery in those given treatment with risedronate before stopping anastrozole.

## Methods

### Study design and participants

**Fig. 1:**
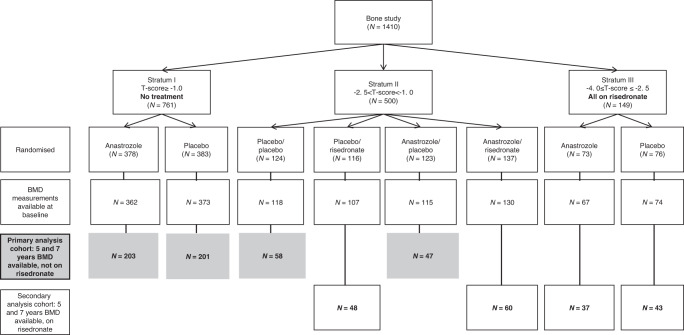
CONSORT diagram. CONSORT diagram for all women in the IBIS-II bone substudy (shaded grey area primary analysis cohort).

The IBIS-II trial recruited 3864 healthy, postmenopausal women at increased risk of breast cancer and randomised them to receive either 1 mg/day anastrozole or matching placebo.^6^ Eligible women were offered the opportunity to enter the bone substudy. One thousand four hundred and ten women entered the bone substudy and were stratified into three groups according to the lowest baseline T-score at either femoral neck or lumbar spine (Fig. 1). Women with healthy T-score (≥−1.0) were entered into stratum I and were monitored only (*N* = 761). Women who were osteopenic (−2.5 ≤ T-score < −1.0) were entered into stratum II and were randomised to receive risedronate (35 mg/week) or matching placebo for five years (*N* = 500). Finally, osteoporotic women with a T-score < −2.5 but greater than −4.0 or those with one to two low trauma fragility fractures (as assessed by spinal radiographs) were entered into stratum III and were required to take risedronate (35 mg/week) (*N* = 149) (Fig. 1). Full inclusion/exclusion criteria for the bone substudy were reported elsewhere.^8^

Our primary analysis (grey shaded boxes in Fig. 1) cohort focuses on women with BMD evaluations at both years 5 and 7 who were randomised to anastrozole or placebo and did not receive risedronate during the five years of active follow-up (stratum I and II). Those who were either randomised or given risedronate for five years (stratum II and stratum III) are referred to as the secondary analysis population (Fig. 1). The trial was approved by the UK North West Multicentre Research Ethics Committee and was done in accordance with the Declaration of Helsinki, under the principles of good clinical practice. Participants provided written informed consent.

### Assessments

All women entering the main IBIS-II study had a dual energy X-ray absorptiometry (DXA) scan prior to study entry, with further assessments by DXA scans at the lumbar spine and total hip at 12, 36, 60 and 84 months. This analysis focuses on the BMD changes between 60 and 84-month follow-up points. At the 60-month follow-up point, participants stopped with their allocated treatment. T-scores have been calculated using either the Lunar^10^ or Hologic^11^ manufacturer’s reference ranges for the lumbar spine (L1–L4) and the NHANES III reference range for the femoral neck region.^12^ All DXA scans were reviewed centrally by two clinical scientists with expertise in bone densitometry (GB and RP) to ensure quality assurance. Both assessors were blinded to randomised treatment allocation.

### Statistical analysis

All analyses were performed on a per protocol basis, in which women were only included if a baseline, 5- and 7-year DXA scan were available. The primary objective was to compare the changes in BMD at the lumbar spine and total hip between anastrozole and placebo between years 5 and 7 in women who were not randomised (osteopenic women in stratum II) or did not receive risedronate (women with health bone density in stratum I). A secondary objective was the same as above comparison but in those who were either randomised (osteopenic women in stratum II) or received risedronate (osteoporotic women in stratum III) for 5 years of follow-up.

All main results are expressed as percent mean BMD changes at the total hip or lumbar spine between 5 and 7 years with corresponding 95% confidence intervals (CI). BMD changes and differences between treatment groups were assessed using *t*-tests for two independent samples with corresponding 95% CI. *P* values were two-sided, based on normal approximation and all confidence intervals were at the 95% level. The Bonferroni correction was applied to account for multiple comparisons. Analyses were performed using STATA version 13.1 (College Station, Texas USA). This trial is registered, number ISRCTN31488319.

## Results

A total of 1410 postmenopausal women were entered into the bone substudy with a median follow-up of 10.8 years (IQR 9.5 to 12.7). Seven hundred and sixty-one women were stratified into stratum I, 500 into stratum II and 149 women into stratum III (Fig. 1). For those who did not receive risedronate (primary analysis cohort (grey shaded boxes)), 509 (52.6%) had baseline, 5- and 7-year DXA scans available. Baseline, 5- and 7-year DXA scans were available for 188 (49.7%) who received risedronate (with or without anastrozole) during the 5 years on trial treatments (Fig. 1).

Baseline characteristics for evaluable women are shown in Table 1 according to analysis cohort and main treatment allocation. In the primary analysis cohort, women in stratum I were significantly younger (*P* < 0.001), had higher body mass index (*P* = 0.003) and significantly high BMD at baseline (*P* < 0.001) compared to those in stratum II. In the secondary analysis cohort, baseline characteristics were evenly distributed apart from baseline BMD, which was significantly higher in women in stratum II (osteopenic) compared to those in stratum III (osteoporotic) (Table 1).

**Table 1:** Baseline characteristics according to stratum and analysis cohort.

	No risedronate (primary analysis cohort)			Risedronate (secondary analysis cohort)		
	Stratum I (*N* = 404)	Stratum II (*N* = 105)	*P* value	Stratum II (*N* = 108)	Stratum III (*N* = 80)	*P* value
Age, median (IQR)	58.6 (54.8–62.2)	60.1 (57.2–64.1)	<0.001	59.7 (55.3–64.8)	61.6 (57.2–64.3)	0.15
BMI (kg/m^2^), median (IQR)	28.5 (25.4–32.6)	26.2 (23.8–30.1)	0.003	25.8 (23.7–29.3)	25.6 (23.4–28.3)	0.61
Previous HRT use (%)	200 (48.7%)	54 (46.2%)	0.63	57 (52.8%)	35 (43.8%)	0.22
Never smokers (%)	227 (55.2%)	74 (63.3%)	0.31	64 (59.3%)	49 (61.3%)	0.38
Hysterectomy (%)	135 (32.9%)	38 (32.5%)	0.94	29 (26.9%)	25 (31.3%)	0.51
Oophorectomy (%)	59 (14.4%)	16 (13.7%)	0.85	20 (18.5%)	6 (7.5%)	0.03
Lowest T-score, median (IQR)	−0.37 (−0.87 to 0.22)	−1.52 (−1.94 to −1.20)	<0.001	−1.63 (−2.02 to −1.23)	−2.75 (−3.0 to −2.5)	<0.001

### Primary analysis cohort: anastrozole vs. placebo in women not on risedronate

#### Stratum I

**Fig. 2:**
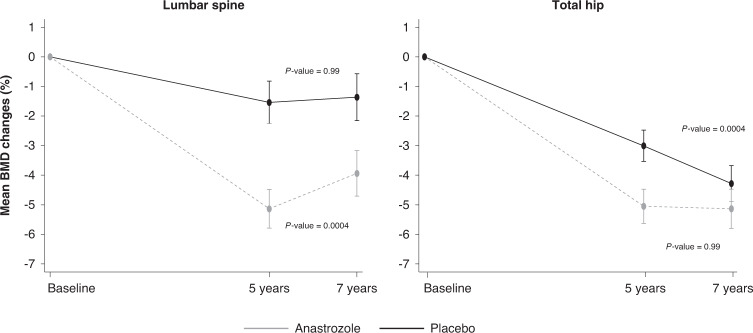
Off-treatment bone mineral density changes (%) with associated 95% confidence intervals between 5 and 7 years at the lumbar spine and total hip for women in stratum I. *P* values refer to comparison between anastrozole vs. placebo for mean BMD changes between 5 and 7-year timepoints.

Five- and seven-year DXA scans were available for analysis in 411 women in stratum I (55.9%), 205 anastrozole vs. 206 placebo). Of these, 14 (3.4%) started with bisphosphonate treatment due to clinicians’ recommendation after 5 years of active follow-up and were excluded from this analysis. Between years 5 and 7, women previously randomised to anastrozole had a significant mean increase in lumbar spine BMD of 1.25% (95% CI 0.73 to 1.77) (*P* = 0.0004). For women on placebo, the mean BMD change during the off-treatment period was only 0.14% (95% CI −0.29 to 0.56; *P* = 0.99) (Fig. 2). Off-treatment differences at the lumbar spine between anastrozole and placebo were statistically different (*P* = 0.0044) (Supplementary Fig. 1). No significant changes in total hip BMD between 5 and 7 years was observed for women previously treated with anastrozole (*P* = 0.99) (Fig. 2). For those on placebo, a continued significant BMD decrease at the total hip of −1.35% (95% CI −1.70 to −0.98; *P* = 0.0004) after treatment cessation was observed (Fig. 2). Changes in BMD between treatment arms s in years 5–7 at the total hip were statistically significant (*P* = 0.0004) (Supplementary Fig. 1). Only one patient randomised to placebo developed osteoporosis by year 7, but she had been osteopenic by year 5 (spinal T-score −2.41).

#### Stratum II

**Fig. 3:**
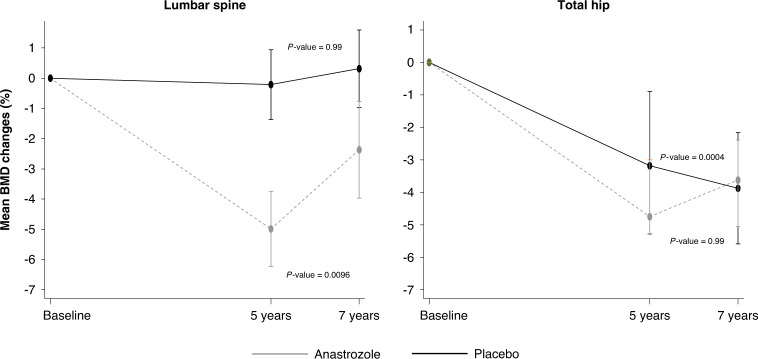
Off-treatment bone mineral density changes (%) with associated 95% confidence intervals between 5 and 7 years at the lumbar spine and total hip for women in stratum II. *P* values refer to comparison between anastrozole vs. placebo for mean BMD changes between 5 and 7-year timepoints.

One hundred and five women (45.1%) had baseline, 5- and 7-year DXA scans available for analysis of which 13 (11.1%) used a bisphosphonate after five years and were excluded. Women previously treated with anastrozole (*N* = 46) had a significant mean BMD increase at the lumbar spine of 2.58% (95% CI 1.03 to 4.14) between years 5 and 7 (*P* = 0.0096) (Fig. 3, Supplementary Fig. 1). For women on placebo (*N* = 58), no significant changes in BMD at the lumbar spine were observed in this time period (0.41% (95% CI −0.55 to 1.37) (*P* = 0.99). Significant differences in BMD changes between women randomised to anastrozole compared to placebo were observed at the lumbar spine (*P* = 0.0284) (Supplementary Fig. 1). At the total hip, a slight increase in BMD (0.44% (95% CI −0.45 to 1.33), *P* = 0.99) for women previously treated with anastrozole was observed, whereas those randomised to placebo showed a continued significant decrease in BMD during the off-treatment period (−1.54% (95% CI −2.39 to −0.69) (*P* = 0.0004) (Fig. 3). The difference in mean BMD changes between the two treatment arms was statistically significant (*P* = 0.012) (Supplementary Fig. 1). Two women developed osteoporosis at the hip and spine, respectively (one in each treatment arm).

### Secondary analysis cohort: anastrozole vs. placebo in women who received risedronate

Two hundred and fifty-three of women in stratum II have been randomised to five years of risedronate (137 anastrozole/risedronate vs. 116 placebo/risedronate). One hundred and eight (45.6%) of them had DXA scans at 5 and 7 years available for analysis. Women previously treated with anastrozole (*N* = 60) had a nonsignificant increase in mean lumbar spine BMD of 1.09% (95% CI 0.23 to 1.96) between 5 and 7 years (*P* = 0.076) (Supplementary Fig. 2). For those on placebo (*N* = 48), a continued nonsignificant decrease in mean lumbar spine BMD change was observed (−1.06% (95% CI −2.11 to −0.02) (*P* = 0.26) (Supplementary Fig. 2). Significant differences between treatment arms at the lumbar spine were observed (*P* = 0.0004). At the total hip, a significant BMD decrease was observed for women randomised to placebo (−1.67 (95% CI 2.46 to −0.88) (*P* = 0.00004) whereas those previously randomised to anastrozole had a small continued nonsignificant decrease of −0.60% (95% CI −1.36 to 0.16) (*P* = 0.48). There were significant differences in BMD changes at the total hip between anastrozole and placebo (*P* = 0.004). A total of six women developed osteoporosis (1 at the total hip, 5 at the lumbar spine), with 4 women on placebo versus 2 randomised to anastrozole).

For osteoporotic women (*N* = 80), a significant increase in lumbar spine BMD was observed for women on anastrozole (1.92% (0.57 to 3.26), *P* = 0.024) whereas a small nonsignificant decrease was seen for women on placebo (−0.54% (95% CI −1.94 to 0.86), *P* = 0.68) (Supplementary Fig. 2). The difference in mean BMD changes between treatment arms was statistically significant (*P* = 0.04). A similar decrease in total hip BMD between 5 and 7 years was observed for anastrozole (−0.30% (95% CI −1.49 to 0.89) and placebo (−0.57% (95% CI −1.49 to 0.34)) (Supplementary Fig. 2).

Table 2 shows overall mean BMD changes in all treatment groups for the overall follow-up period (between baseline and 7 years). Overall, significant differences decrease in mean lumbar spine BMD at 7 years were observed for women on anastrozole compared to placebo in stratum I (−3.94% vs. −1.37%, *P* = 0.0007) (Table 2). Thirty women in stratum I developed osteopenia after 5 years on anastrozole, of which nine had an improvement in BMD after stopping with treatment. Similar decrease in total hip BMD was observed between anastrozole and placebo after 7 years of follow-up (−5.06% vs. −4.31%, *P* = 0.7) (Table 2). This translates to seven women developing osteopenia at the hip on anastrozole, of which two showed an improvement after stopping with treatment. In osteopenic women (stratum II) who were randomised to placebo, a nonsignificant improvement in BMD was observed at the lumbar spine (*P* = 0.175) and a significant increase at the total hip (*P* = 0.0007) in women randomised to risedronate compared to those who did not receive risedronate (Table 2). In osteopenic women randomised to anastrozole, the addition of risedronate significantly improved BMD at the lumbar spine (*P* = 0.0007) but not at the total hip where change sin BMD were similar between those received risedronate or not (*P* = 0.99) (Table 2). Eight women with osteopenia at baseline who were randomised to anastrozole developed osteoporosis over 5 years (4 on risedronate and 4 on placebo). Finally, in osteoporotic women (stratum III), the addition of risedronate did not have any impact on BMD changes between baseline and 7 years, irrespective of main randomisation (Table 2).

**Table 2:** BMD changes at the lumbar spine and total hip with associated 95% confidence intervals between baseline and 7 years according to strata and treatment allocation.

	Stratum I			Stratum II						Stratum III		
	P (*N* = 206)	A (*N* = 205)	*P* value	P/P (*N* = 64)	P/R (*N* = 48)	*P* value	A/P (*N* = 53)	A/R (*N* = 60)	*P* value	P (*N* = 43)	A (*N* = 37)	*P* value
Lumbar spine	−1.37 (−2.16 to −0.57)	−3.94 (−4.71 to −3.17)	0.0007	0.32 (−0.97 to 1.60)	2.83 (0.92 to 4.74)	0.175	−2.37 (−3.97 to −0.77)	1.34 (−0.11 to .78)	0.0007	3.80 (1.70 to 5.90)	2.82 (0.91 to 4.74)	0.99
Total hip	−4.31 (−4.91 to −3.71)	−5.06 (−5.72 to −4.40)	20.7	−3.85 (−4.87 to −2.83)	−0.31 (−1.74 to 1.12)	0.0007	−3.66 (−5.02 to −2.31)	−2.44 (−3.76 to −1.12)	0.99	−0.17 (−1.61 to 1.27)	−0.86 (−2.74 to 1.03)	0.99

Women in stratum I had healthy BMD at baseline, those in stratum II were osteopenic, and those in stratum III were osteoporotic. *P* values are for a comparison between allocated treatment groups. *P* values are corrected for multiple comparisons.

A Anastrozole, *P* Placebo, *R* Risedronate.

## Discussion

The IBIS-II bone substudy has previously shown that 5 years of anastrozole used for prevention of breast cancer in postmenopausal women at increased risk of developing the disease was associated with a greater BMD loss at the lumbar spine and total hip for women with normal bone health at entry or those with osteopenia compared to those randomised to placebo.^8^ Here we showed that 2 years after stopping treatment with 1 mg/day anastrozole for 5 years BMD at the lumbar spine improved whereas BMD at the total hip stabilised over the same time period in women who did not receive any bisphosphonates. We also demonstrated that the improvements in BMD at the lumbar spine were significantly more pronounced for those originally randomised to anastrozole compared with those on placebo. There was furthermore evidence of partial recovery of total hip BMD for those on anastrozole, with no further decrease in BMD at this site once anastrozole treatment was stopped. We also observed an improvement of BMD at the lumbar spine for osteopenic and osteoporotic women if they received bisphosphonates during the trial, but no improvements were observed at the total hip for this subgroup of women.

Following completion of endocrine therapy, anastrozole withdrawal was associated with an increase in BMD. This is probably due to an increase of oestrogen levels and hence a decrease in bone resorption.^5^ At the total hip, we found a stabilisation of BMD for women who were originally randomised to anastrozole whereas those on placebo showed a continued BMD loss at the total hip. This is most likely explained by the fact that age-related BMD loss has been prevented by an increase in oestrogen levels after stopping the aromatase inhibitor. Oestrogen plays an important role in determining bone loss in postmenopausal women and is strongly linked to cortical bone loss.^13^ Overall, the magnitude of BMD loss during the active treatment period in our study is similar to previous trials investigating an aromatase inhibitor in the adjuvant^14–16^ or preventive setting.^5^ The Arimidex Tamoxifen Alone or in Combination (ATAC) trial compared the effectiveness of anastrozole to tamoxifen in postmenopausal women.^1^ Bone mineral density at the lumbar spine and total hip was measured after 5 years of treatment with anastrozole and then 1 and 2 years after stopping treatment.^17^ In this trial, anastrozole-related bone loss did not continue into the off-treatment period (2 years after stopping allocated treatment). Furthermore, there was a recovery of lumbar spine BMD of 4.0% and absence of further BMD decline at the total hip (increase of 0.5%). In the second adjuvant study investigating BMD changes after treatment cessation, similar improvements were observed.^18^ The results from the adjuvant setting are very similar to our findings in the preventive setting described here.

The clinical significance of a relative improvement at the lumbar spine and total hip BMD of 1.25% after stopping anastrozole indicates only a partial recovery at both sites. If we had measured BMD for all participants after a further 3 years, then there may have been a full recovery but that is unlikely. When oestrogen is administered to a postmenopausal woman the increase in BMD tends to be greatest in the first 2 years and then reaches a plateau.^19^ Our results showed no further BMD loss and therefore there should be no further increase in the risk of fracture in these postmenopausal women.

The only other study investigating an aromatase inhibitor in the preventive setting was the MAP.3 trial.^4^ The nested bone substudy protocol of the MAP.3 trial^5^ concentrated on the examination of the safety of exemestane on bone health, focusing on bone density and structure. They found that 2 years of exemestane treatment worsened bone loss in postmenopausal women despite calcium and vitamin D supplementation. However, they were not able to assess long-term effects of exemestane on BMD and therefore a direct comparison between the two prevention studies is not possible.

The changes after stopping risedronate alone were similar to those described previously. Watts et al.^20^ reported the changes in BMD after stopping a 3-year course of risedronate 5 mg/day in 293 women with postmenopausal osteoporosis. They found that over a year there was a decrease in lumbar spine (0.8%) and femoral neck (1.2%). These were slightly larger than observed in the present study of 0.5% over 2 years at the lumbar spine. Overall, BMD loss might be expected to be greater at the lumbar spine than the total hip as it is more metabolically active, but in older women the development of spinal osteoarthritis can mask bone loss. The changes in the untreated group in our study were similar to BMD changes reported in women more than 10 years since the menopause who have an annual rate of bone loss from the spine of 0.72% and the total hip of 0.75% per year^21^ compared to 1.35% over 2 years in our study.

A strength of this study is that this is the only analysis looking at the off-treatment effects on BMD changes in the breast cancer prevention setting. We included a large proportion of women with normal BMD or osteopenia, with long-term follow-up (7 years). Women included in this analysis come from a large prevention study with excellent clinical records and good quality controls for DXAs. For the primary objective, only women who were either randomised to or did not receive risedronate were included. Limitations of our study include that we did not have a complete DXA data set for all follow-up timepoints (for our primary analysis set 509/1008 (50.5%)). The main reason for noncomplete datasets were withdrawal from the main study in most cases due to anastrozole-related side effects. However, we only included women in this analysis who had a full set of DXA scans (baseline, 5 and 7 years) and hence all women finished their allocated treatment. Therefore anastrozole-induced BMD changes during this time period are the true effect and no selection bias was introduced. A small number of women were treated with bisphosphonates after 5 years, but we cannot be sure whether this represents the true number of women who have taken a bisphosphonate and therefore this may introduce some bias.

In conclusion, this study is the first to quantify the long-term impact on BMD in women at increased risk of developing breast cancer. Bone loss associated with anastrozole use is partially reversible after stopping treatment, particularly at the lumbar spine. For patients who have been given risedronate for osteoporosis prevention, bone mineral density improved only at the lumbar spine but not the total hip. Anastrozole-related bone loss seems to be manageable and any risk to bone health should be weighed against overall efficacy and tolerability for the preventive treatment of at high-risk women.

## Supplementary information

Supplemental material

## Data Availability

Data will be available according to IBIS-II’s data-sharing plan. Request for specific analyses or data can be submitted via email to j.cuzick@qmul.ac.uk. Details for data-sharing policy and application process can be found at www.qmul.ac.uk/wolfson/centres/ccp/data-sharing.

## References

[1] Cuzick J, Sestak I, Baum M, Buzdar A, Howell A, Dowsett M (2010). Effect of anastrozole and tamoxifen as adjuvant treatment for early-stage breast cancer: 10-year analysis of the ATAC trial. Lancet Oncol..

[2] Coombes RC, Kilburn LS, Snowdon CF, Paridaens R, Coleman RE, Jones SE (2007). Survival and safety of exemestane versus tamoxifen after 2-3 years’ tamoxifen treatment (Intergroup Exemestane Study): a randomised controlled trial. Lancet.

[3] Goss PE, Ingle JN, Martino S, Robert NJ, Muss HB, Piccart MJ (2003). A randomized trial of letrozole in postmenopausal women after five years of tamoxifen therapy for early-stage breast cancer. N. Engl. J. Med..

[4] Goss PE, Ingle JN, Ales-Martinez JE, Cheung AM, Chlebowski RT, Wactawski-Wende J (2011). Exemestane for breast-cancer prevention in postmenopausal women. N. Engl. J. Med..

[5] Cheung AM, Tile L, Cardew S, Pruthi S, Robbins J, Tomlinson G (2012). Bone density and structure in healthy postmenopausal women treated with exemestane for the primary prevention of breast cancer: a nested substudy of the MAP.3 randomised controlled trial. Lancet Oncol..

[6] Cuzick J, Sestak I, Forbes JF, Dowsett M, Knox J, Cawthorn S (2014). Anastrozole for prevention of breast cancer in high-risk postmenopausal women (IBIS-II): an international, double-blind, randomised placebo-controlled trial. Lancet.

[7] Cuzick J, Sestak I, Forbes JF, Dowsett M, Cawthorn S, Mansel RE (2020). Use of anastrozole for breast cancer prevention (IBIS-II): long-term results of a randomised controlled trial. Lancet.

[8] Sestak I, Singh S, Cuzick J, Blake GM, Patel R, Gossiel F (2014). Changes in bone mineral density at 3 years in postmenopausal women receiving anastrozole and risedronate in the IBIS-II bone substudy: an international, double-blind, randomised, placebo-controlled trial. Lancet Oncol..

[9] Sestak I, Blake GM, Patel R, Coleman RE, Cuzick J, Eastell R (2019). Comparison of risedronate versus placebo in preventing anastrozole-induced bone loss in women at high risk of developing breast cancer with osteopenia. Bone.

[10] GE Medical systems. *DPX Series Operator’s Manual*. GE Healthcare, 1998.

[11] Kelly, T. L. Bone mineral density reference databases for American men and women. *J. Bone Mine. Res*. **5**((Suppl 1), S249 (1990).

[12] Looker AC, Wahner HW, Dunn WL, Calvo MS, Harris TB, Heyse SP (1998). Updated data on proximal femur bone mineral levels of US adults. Osteoporos. Int..

[13] Khosla S, Melton LJ, Riggs BL (2011). The unitary model for estrogen deficiency and the pathogenesis of osteoporosis: is a revision needed?. J. Bone Min. Res..

[14] Coleman RE, Banks LM, Girgis SI, Kilburn LS, Vrdoljak E, Fox J (2007). Skeletal effects of exemestane on bone-mineral density, bone biomarkers, and fracture incidence in postmenopausal women with early breast cancer participating in the Intergroup Exemestane Study (IES): a randomised controlled study. Lancet Oncol..

[15] Eastell R, Adams JE, Coleman RE, Howell A, Hannon RA, Cuzick J (2008). Effect of anastrozole on bone mineral density: 5-year results from the anastrozole, tamoxifen, alone or in combination trial 18233230. J. Clin. Oncol..

[16] Hadji P, Asmar L, van Nes JG, Menschik T, Hasenburg A, Kuck J (2011). The effect of exemestane and tamoxifen on bone health within the Tamoxifen Exemestane Adjuvant Multinational (TEAM) trial: a meta-analysis of the US, German, Netherlands, and Belgium sub-studies. J. Cancer Res. Clin. Oncol..

[17] Eastell R, Adams J, Clack G, Howell A, Cuzick J, Mackey J (2011). Long-term effects of anastrozole on bone mineral density: 7-year results from the ATAC trial. Ann. Oncol..

[18] Coleman RE, Banks LM, Girgis SI, Vrdoljak E, Fox J, Cawthorn SJ (2010). Reversal of skeletal effects of endocrine treatments in the Intergroup Exemestane Study. Breast Cancer Res. Treat..

[19] Cauley JA, Robbins J, Chen Z, Cummings SR, Jackson RD, LaCroix AZ (2003). Effects of estrogen plus progestin on risk of fracture and bone mineral density: the Women’s Health Initiative randomized trial. JAMA.

[20] Watts NB, Chines A, Olszynski WP, McKeever CD, McClung MR, Zhou X (2008). Fracture risk remains reduced one year after discontinuation of risedronate. Osteoporos. Int..

[21] Gossiel F, Altaher H, Reid DM, Roux C, Felsenberg D, Gluer CC (2018). Bone turnover markers after the menopause: T-score approach. Bone.

