# Supplementary figures and images for: Off-treatment bone mineral density changes in postmenopausal women receiving anastrozole for 5 years: 7-year results from the IBIS-II prevention trial
Source: Br J Cancer. 2021 Jan 22;124(8):1373–8. doi: 10.1038/s41416-020-01228-2 (PMC8039042; doi:10.1038/s41416-020-01228-2)

**Supplementary material**


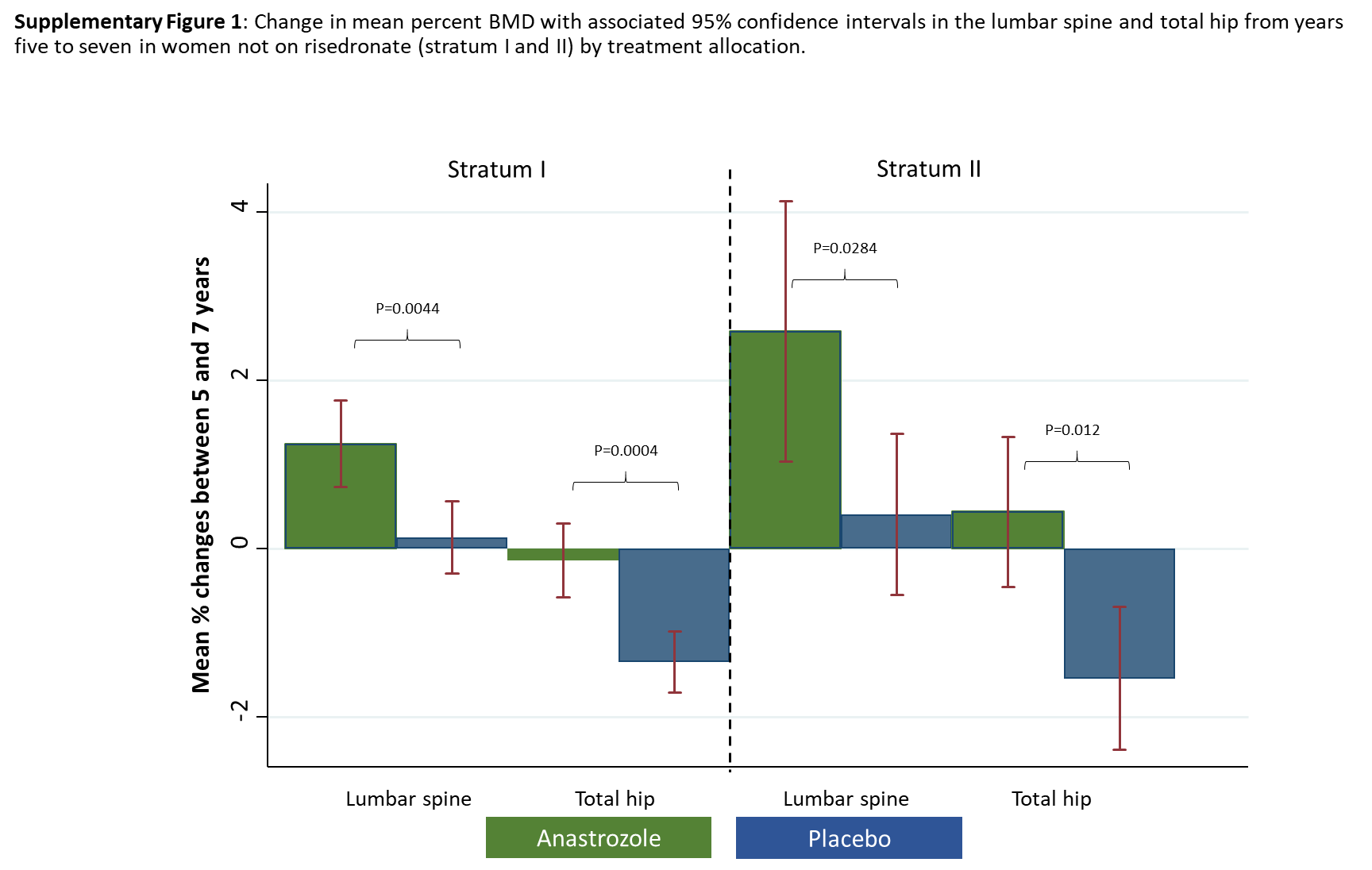


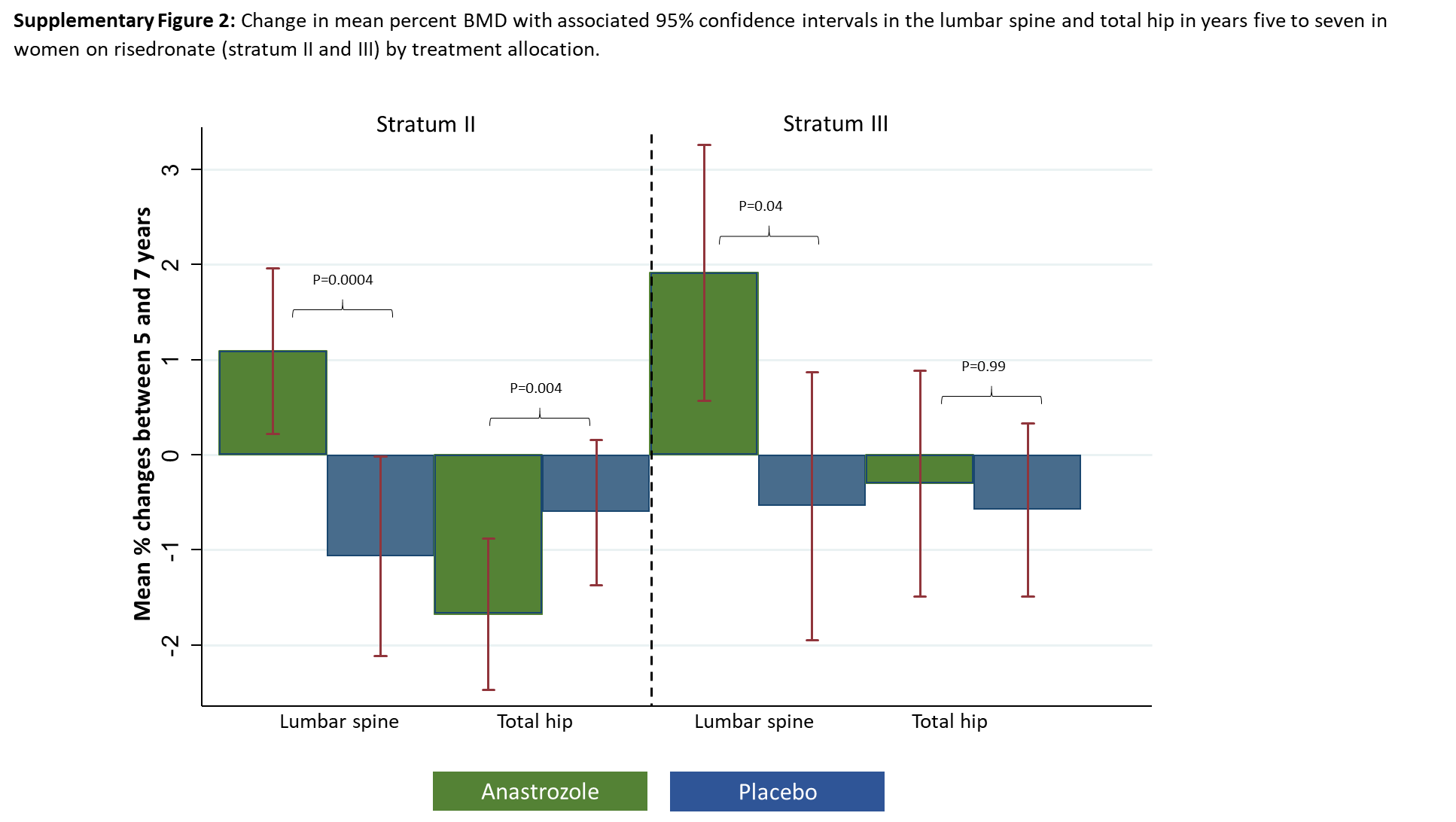

Supplement: Supplementary file 1 — Supplemental material [file 41416_2020_1228_MOESM1_ESM.docx]
